# Ursodeoxycholic acid for the prevention of symptomatic gallstone disease after bariatric surgery: statistical analysis plan for a randomised controlled trial (UPGRADE trial)

**DOI:** 10.1186/s13063-020-04605-7

**Published:** 2020-07-23

**Authors:** Sylke Haal, Maimoena S. S. Guman, L. Maurits de Brauw, Ruben N. van Veen, Ruben Schouten, Paul Fockens, Victor E. A. Gerdes, Marcel G. W. Dijkgraaf, Rogier P. Voermans

**Affiliations:** 1 Department of Gastroenterology and Hepatology, Amsterdam Gastroenterology Endocrinology Metabolism, Amsterdam UMC, University of Amsterdam, Amsterdam, the Netherlands; 2 Department of Internal Medicine, Spaarne Gasthuis, Hoofddorp, the Netherlands; 3 Department of Internal and Vascular Medicine, Amsterdam UMC, University of Amsterdam, Amsterdam, the Netherlands; 4 Department of Surgery, Spaarne Gasthuis, Hoofddorp, the Netherlands; 5 Department of Surgery, OLVG, Amsterdam, the Netherlands; 6 Department of Surgery, Flevoziekenhuis, Almere, the Netherlands; 7 Department of Epidemiology and Data Science, Amsterdam UMC, University of Amsterdam, Amsterdam, the Netherlands

**Keywords:** Bariatric surgery, Symptomatic gallstone disease, Ursodeoxycholic acid, Randomised controlled trial, Statistical analysis plan

## Abstract

**Background:**

Approximately 8–15% of patients undergoing bariatric surgery develop symptomatic gallstone disease within 24 months after surgery. Ursodeoxycholic acid (UDCA) seems to effectively prevent the formation of gallstones detectable by ultrasound after bariatric surgery. The aim of the UPGRADE trial is to provide evidence on the prophylactic use of UDCA in preventing symptomatic gallstone disease postoperatively.

**Methods:**

The UPGRADE trial is designed as a randomised, placebo-controlled, double-blind multicentre trial in patients with morbid obesity undergoing Roux-en-Y gastric bypass (RYGB) or sleeve gastrectomy (SG). Patients are randomly assigned to either UDCA 900 mg daily for 6 months or placebo treatment. This paper details the statistical analysis plan (SAP) of this trial and was submitted before outcome data were available.

**Results:**

The primary endpoint of this trial is symptomatic gallstone disease within 24 months after bariatric surgery, defined as admission or hospital visit for symptomatic gallstone disease. Secondary outcomes consist of the development of gallstones/sludge on ultrasound at 24 months in the gallstone-negative group at baseline, presence of gallstones/sludge on ultrasound at 24 months, number of cholecystectomies, side effects of UDCA, therapy compliance, quality of life, costs and revenues. Analyses will be completed according to this pre-specified SAP. The main analysis will be performed as a standard ITT analysis using the chi-squared test.

**Discussion:**

The UPGRADE trial will show if prophylactic use of UDCA reduces the incidence of symptomatic gallstone disease after bariatric surgery. Unforeseen deviations from the SAP at the time of analysis will be motivated and discussed.

**Trial registration:**

The Netherlands Trial Register NL5954. Registered on 21 November 2016.

## Introduction

The number of bariatric interventions for morbid obesity is increasing worldwide. Rapid weight loss is a major risk factor for gallstone development. Approximately 8–15% of patients undergoing bariatric surgery develop symptomatic gallstone disease within 2 years after surgery. Prophylactic administration of ursodeoxycholic acid (UDCA), an oral bile acid, can prevent the formation of gallstones after bariatric surgery by reducing bile lithogenicity [1–3]. However, little evidence is available regarding the efficacy of UDCA in preventing symptomatic gallstone disease. The UPGRADE trial (Ursodeoxycholic acid for the prevention of symptomatic gallstone disease after Roux-en-Y gastric bypass and sleeve gastrectomy) is designed to investigate whether UDCA reduces symptomatic gallstone disease after bariatric surgery. The trial protocol was previously published [4], and the present paper is the proposed statistical analysis plan (SAP). This SAP is in line with the corresponding reporting guideline for SAPs [5] and was written without knowledge of the outcomes by the investigators.

## Summary study protocol

The UPGRADE trial is a randomised, placebo-controlled, double-blind multicentre trial comparing the prophylactic use of UDCA versus placebo in patients with morbid obesity undergoing bariatric surgery. Patients with an intact gallbladder and scheduled to undergo Roux-en-Y gastric bypass (RYGB) or sleeve gastrectomy (SG) were included. Patients were excluded in case of the existence of symptomatic gallstone disease prior to bariatric surgery, prior bariatric surgery, prior gallbladder surgery, inflammatory bowel disease and other conditions of the small intestine and liver which may interfere with enterohepatic circulation of bile salts, hypersensitivity to ingredients of UDCA or placebo, and intake of UDCA within the last 30 days before screening. Patients were randomly assigned to either UDCA 900 mg daily for 6 months or placebo in a 1:1 ratio. Randomisation was done prior to surgery with an online computer software program (ALEA AMC, Amsterdam, the Netherlands, version 2.2) and implemented into a web-based application. Randomisation was stratified for the presence of gallstones (yes versus no) and type of surgery (RYGB versus SG) and performed by using random block sizes of 4, 6 and 8. Data are collected during hospitalisation and study visits at 6 weeks (window 4–8 weeks) and 16 weeks (window 14–18 weeks) and at 6 months (window 5–9 months), 12 months (window 10–15 months) and 24 months (window 24–30 months) by standardised case report forms. Questionnaires are gathered at baseline, 6 weeks, and 3, 6, 12, 18 and 24 months (windows 1 week before to 2 weeks after the date of administration). The primary outcome is symptomatic gallstone disease within 24 months, defined as admission or hospital visit for symptomatic gallstone disease. Secondary outcomes consist of the development of gallstones/sludge on ultrasound at 24 months in the gallstone-negative group at baseline (window 18–30 months), presence of gallstones/sludge on ultrasound at 24 months (window 18–30 months), number of cholecystectomies, time to symptomatic gallstone disease and cholecystectomy, side effects of UDCA, therapy compliance, quality of life, costs and revenues. Safety outcomes are adverse events (AEs), serious adverse events (SAEs) and suspected unexpected serious adverse reactions (SUSARs). For the sample size calculation, we refer to the study protocol [4]. On 22 October 2018, all included patients underwent surgery, and complete 2-year follow-up data are expected in November 2020. For the complete study protocol, we refer to the previous publication [4].

### Protocol developments

The UPGRADE is registered at the Netherlands Trial Register (NL5954) on 21 November 2016. The institutional review board (IRB) of the Slotervaart Hospital and Reade, Amsterdam, approved the protocol on 9 November 2016. The trial is conducted in three high-volume bariatric centres. On 18 July 2017, the IRB approved a protocol amendment in which the bariatric procedure SG was added to the inclusion criteria, because of increasing use worldwide, in Europe and in participating centres [6]. The initial study protocol stated that patients had to start the trial medication as soon as possible, but no later than 2 weeks after surgery. We extended the accepted start of trial medication during the initial course of the trial, in case of difficulties taking the trial medication within the first 2 weeks after surgery. Patients were still instructed to start the trial medication as soon as possible, preferably within 2 weeks, but 8 weeks at the latest. Additionally, the definition of some outcome measurements was slightly adapted. The IRB approved all above-mentioned protocol changes. No changes were made regarding the sample size.

## Statistical analysis plan

### General principles

The randomisation code will not be broken until the last patient reaches 24 months of follow-up, all primary endpoints are reviewed by a blinded independent endpoint adjudication committee, and data cleaning is completed. The analyses will be performed after data verification and validation have been carried out and after this SAP has been accepted for publication. Analyses will be done by the investigators and biostatistician of the UPGRADE study group, using the most recent version of the SPSS statistical software at the time of analysis. All statistical tests will use a two-sided *p* value of 0.05, and two-sided 95% confidence intervals will be presented for all estimates.

### Patient flow diagram

**Fig. 1:**
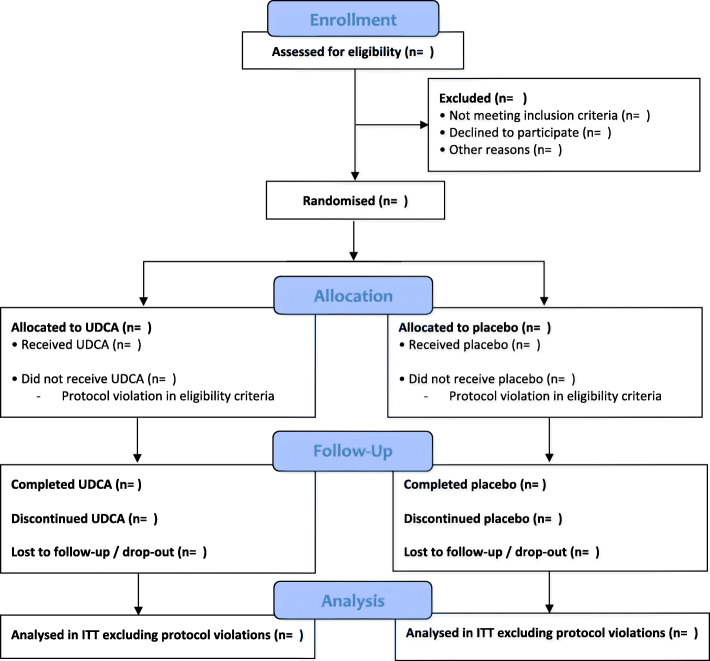
Participants flow diagram

A flow diagram of study participants will be displayed in line with the Consolidation Standard of Reporting Trials (CONSORT) recommendations and finalised upon external peer review (Fig. 1) [7].

### Treatment according to protocol and withdrawal

Treatment was regarded according to the study protocol when a patient started the trial medication within 2 weeks after surgery, but 8 weeks at the latest. A maximum mid-term break of 4 weeks was allowed during the treatment course, and good therapy compliance was defined as a minimum of 300 pills taken within a time frame of 6–8 months (> 80%). Patients were also considered as treated per protocol when the trial medication was terminated early due to reaching the primary endpoint or death. The numbers of losses to follow-up (withdrawal from follow-up) and dropouts (withdrawal from intervention) will be summarised by study arm.

### Definition of intention-to-treat, modified intention-to-treat, full analysis sets and per-protocol sets

**Fig. 2:**
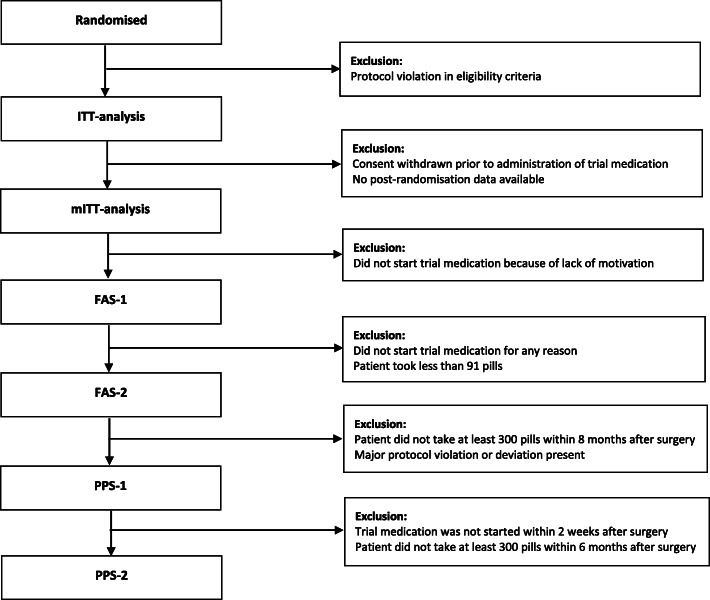
Summary of participant inclusion and exclusion for ITT, mITT, FAS and PPS

The main analysis will follow the intention-to-treat (ITT) principle with all patients analysed in their randomisation group, irrespective of protocol adherence. Only patients with a protocol violation concerning eligibility are excluded from the ITT analysis. Protocol violation in eligibility refers to randomised patients who did not fulfil inclusion criteria or randomised patients who did meet an exclusion criterion (e.g. patients who did not undergo bariatric surgery). Patients receiving SG instead of RYGB before the approval date of the protocol amendment are also considered protocol violations in eligibility. The modified ITT analysis (mITT) will include all patients except for patients excluded from the ITT and patients without a post-randomisation measurement (e.g. patients who withdrew consent prior to administration of the trial medication or patients immediately lost to follow-up). In addition, two full analysis sets (FASs) of subjects will be made. In the first FAS, all patients will be included except for patients excluded in the mITT and patients who never commenced trial medication because of lack of motivation. Thus, patients with severe postoperative complications who were not able to start the trial medication within 8 weeks postoperatively will be included. In the second FAS, all patients will be included except for patients who were excluded in the first FAS, patients who never commenced trial medication for any reason and patients who took less than 91 pills (25% of the total amount of trial medication). We will also use two per-protocol sets (PPSs) of subjects. The first PPS will include patients who started the trial medication within 8 weeks after surgery and took a minimum of 300 pills (> 80%) within 8 months after surgery. The second PPS will include patients who started the trial medication within 2 weeks after surgery and took a minimum of 300 pills (> 80%) within 6 months after surgery. Both sets will only include patients without major protocol violations and deviations. A summary for participant inclusion and exclusion for ITT, mITT, FAS and PPS will be displayed in Fig. 2. The number of participants per data set will be displayed in supplemental Fig. 1.

### Patient replacement and handling of missing data

Patients not fulfilling eligibility criteria resulting in the exclusion of the ITT analysis will be replaced. If outcome data cannot be obtained by a study follow-up visit at the outpatient clinic, the patient will be contacted by telephone to gather primary and most secondary outcome data. The postoperative ultrasound of these patients and all data on patients that cannot be contacted will be considered missing. For missing data, a multiple (% of missing data) imputation approach will be selected and justified at the time of analysis, based on the observed missing data pattern and using relevant patient characteristics and outcome predictors. The imputed values will be primarily estimated within the observed data ranges. We will not impute missing values if the percentage missing is below 2. If the missing data appear not missing at random, a worse- and best-case scenario will be run, in which all patients lost to follow-up in the placebo arm are considered free of symptomatic gallstone disease, and all patients lost to follow-up in the UDCA arm are considered not free of symptomatic gallstone disease, and vice versa. Missing values of baseline characteristics will not be imputed. Mixed effects modelling will be applied for the analysis of repeated measures from questionnaire data, thereby simultaneously accounting for missing data.

### Baseline characteristics

The following characteristics of patients will be shown per treatment arm in a baseline table (Table 1): age (years), sex (% F), ethnicity, weight at baseline and at time of surgery (kg), body mass index (BMI) (kg/m^2^) at baseline, hypertension (% yes), dyslipidaemia (% yes), type 2 diabetes (% yes), pain syndrome (% yes), history of abdominal surgery (% yes), medication prior to surgery (% statins, birth control pills, oestrogens), preoperative cholecystolithiasis (% yes), type of surgery (% RYGB), baseline health utility score (derived from the EuroQol 5 Dimensions 5 Level (EQ-5D-5L) questionnaire and the corresponding Dutch scoring algorithm for observed health status profiles) [8]. Categorical variables will be summarised as the proportion of patients with the count divided by the total number of evaluated patients. Continuous variables with a normal distribution will be summarised using means and standard deviations, whereas medians and interquartile ranges will be used in case of non-normal distributions. For continuous variables, a footnote will be added to show the number of evaluated patients. Differences between the study arms will be analysed using the chi-squared test, independent *t* test or Mann-Whitney test and reported at the discretion of the publishing journal’s policy regarding significance testing in baseline tables for RCTs.

**Table 1:** Baseline characteristics

	UDCA (***n***=)	Placebo (***n***=)
**Age**—years, mean (SD)		
**Sex**—F, *n* (% *n*/*N*)		
**Ethnicity**		
Dutch		
Surinamese		
Dutch Caribbean		
Moroccan		
Turkish		
Others		
**Baseline weight**—kg		
**Baseline BMI**—kg/m^2^		
**Hypertension**—yes, *n* (% *n*/*N*)		
**Dyslipidaemia**—yes, *n* (% *n*/*N*)		
**Type 2 diabetes**—yes, *n* (% *n*/*N*)		
**Pain syndrome**—yes, *n* (% *n*/*N*)		
**History of abdominal surgery**—yes, *n* (% *n*/*N*)		
**Medication prior to surgery**—yes, *n* (% *n*/*N*)		
Statins		
Birth control pills		
Oestrogen		
**Preoperative cholecystolithiasis**—yes, *n* (% *n*/*N*)		
**Type of surgery**—RYGB, *n* (% *n*/*N*)		
**Baseline health utility score**—*n*, mean (SD)		

### Assessment and analysis of primary outcome

The primary outcome is the proportion of patients with symptomatic gallstone disease within 24 months of follow-up. Symptomatic gallstone disease is defined as a biliary disease (biliary pancreatitis, acute cholecystitis, choledocholithiasis, cholangitis or biliary colics) for which a hospital visit or admission was required. To assess the primary outcome, follow-up appointments are held at 6 and 16 weeks, and 6, 12 and 24 months by one of the blinded investigators. All primary endpoints will be reviewed by a blinded independent endpoint adjudication committee.

To provide an estimator of the effectiveness of UDCA, we will perform a standard ITT analysis. The difference between outcomes by study arm will be compared using the chi-squared test with a risk ratio (unless editors of the accepting journal suggest to report odds ratio) as an indicator for effect size, as shown in Table 2. The results of the mITT and other data sets will be provided in a supplementary appendix, if no contradictory findings are to be reported.

**Table 2:** Gallstone-related outcomes

	UDCA (***n***=)	Placebo (***n***=)	Effect size	***p***
*Primary outcome*				
**Symptomatic gallstone disease**—*n* (% *n*/*N*)				
Biliary pancreatitis				
Acute cholecystitis				
Choledocholithiasis				
Cholangitis				
Biliary colic				
*Secondary outcomes*				
**Presence of gallstones/sludge on ultrasound at 24 months**—*n* (% *n*/*N*)				
**Newly formed gallstones/sludge on ultrasound at 24 months**—*n* (% *n*/*N*)				
**Cholecystectomy**—*n* (% *n*/*N*)				

### Assessment and analysis of secondary outcomes

The assessment and analysis of the secondary outcomes are described separately for each outcome.

#### Time to symptomatic gallstone disease

The time to symptomatic gallstone disease will be compared using Kaplan-Meier survival analysis. The outcome will be provided in the text.

#### The presence of gallstones/sludge in patients on ultrasound at 24 months

The date and result of the ultrasound at 24 months (window 18–30 months) will be recorded in a case report form. The numbers of patients in both study arms with ultrasounds performed and with gallstones at 24 months will be reported. The chi-squared test results and preferred measure for effect size will be shown in Table 2.

#### Newly formed gallstones/sludge on ultrasound in patients on ultrasound at 24 months

In patients without gallstones at baseline, the total number of patients with newly formed gallstones on ultrasound at 24 months will be reported. The chi-squared test results and preferred measure for effect size will be shown in Table 2.

#### The number of and time to cholecystectomies

The total number of patients with cholecystectomies in both study arms will be reported. Whether a patient underwent a cholecystectomy is registered by an investigator in the case report form and verified at the 24-month follow-up. The chi-squared test will be used to compare the outcomes between the study arms. The time to cholecystectomy will be compared using Kaplan-Meier survival analysis. The number of cholecystectomies and preferred effect size will be shown in Table 2, and the outcome of the Kaplan-Meier survival will be provided in the text.

#### Therapy compliance

Therapy compliance is measured in three ways. Patients are asked for therapy compliance at their 6-month follow-up appointment to indicate how many days per week they took the trial medication. Secondly, patients are asked to return the package material of the trial medication that was taken together with any leftovers of the trial medication. Consequently, a pill count is performed by the clinician. Furthermore, patients have to complete an online questionnaire about trial medication adherence 6 weeks and 6 months after surgery. The questionnaire consists of a modified eight-item Morisky Medication Adherence Scale. Questions address the method of taking the trial medication, difficulties in taking the trial medication, potential solutions for taking the trial medication and the reason for discontinuing the trial medication prematurely. Therapy compliance will be defined in 5 categories: did not use trial medication, poor (< 91 pills (25%)), moderate (< 300 pills (< 80%)), good (≥ 300 pills (> 80%)) and complete therapy (all pills = 364 (100%)). The Mantel-Haenszel chi-squared test will be used to compare therapy compliance of UDCA and placebo, and the outcome will be shown in a secondary outcome table (Table 3). The evaluation of the questionnaires about the trial medication will not be included in this SAP and primary paper.

**Table 3:** Therapy compliance, side effects and SAEs

	UDCA (***n***=)	Placebo (***n***=)	***p***
**Therapy compliance**—*n* (% *n*/*N*)			
Did not start trial medication			
Poor (< 91 pills)			
Moderate (< 300 pills)			
Good (≥ 300 pills)			
Complete therapy (364; all pills)			
**Side effects of treatment (AEs)**—*n* (% *n*/*N*)			
Diarrhoea			
Abdominal pain			
Skin rash			
**SAEs**—*n* (% *n*/*N*)			
Related to trial medication			
Not related to trial medication			

#### Quality of life and health utility

**Fig. 3:**
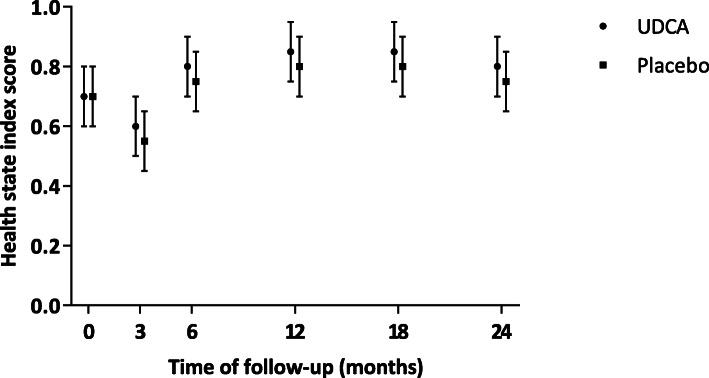
Example health utility scores over time

The 36-Item Short Form Health Survey and/or RAND 36-item Health Survey will be used to gather information on patients’ health-related quality of life status at baseline and 12 and 24 months. These questionnaires are gathered during regular follow-up of bariatric patients independent of participation in a trial. To minimise the burden of participation in the UPGRADE trial, these questionnaires were not administered doubly. Hence, changes in quality of life will only be reported and provided in a supplementary appendix for a convenience sample of measurements. The EQ-5D-5L will be used to gather information on patients’ health utility at baseline and 3, 6, 12, 18 and 24 months. Based on the EQ-5D-5L scoring profiles, utility scores over time will be derived from a readily available scoring algorithm for the Netherlands [8]. The follow-up data will be assessed by repeated measurement analysis with baseline data as a covariate, using a generalised linear mixed model based on the observed correlation matrix amongst successive measurements. The baseline health utility score will be provided in the baseline table, and the utility scores over time will be displayed per treatment arm in a figure (Fig. 3; with baseline data included for reference).

#### Cost-effectiveness, cost-utility and budget impact analyses

The economic evaluation of prophylactic UDCA use after bariatric surgery will be performed as cost-effectiveness analyses and cost-utility analyses from a societal perspective with the costs per patient without symptomatic gallstone disease and the costs per quality-adjusted life year as primary economic outcomes. Also, a budget impact analysis from a governmental, insurer and health care provider perspective will be performed describing the financial consequences of prophylactic use of UDCA and reduced number of surgical interventions for the health care budgets of intramural specialised care, extramural use of drugs, general practitioner care and paramedic care. The economic evaluation will not be included in this SAP.

### Assessment and analysis of safety outcomes and side effects

Safety outcomes are AEs, SAEs and SUSARs. Because AEs are very common after bariatric surgery, we only recorded the AE if there was a reasonable suspicion of the AE being an effect of the trial medication and in case the AE was graded at least as moderately severe or mildly severe lasting longer than 1 week (Table 3). SAEs after bariatric surgery are relatively common as well, so only SAEs that occurred after the start of the trial medication were recorded (Table 3). The number of trial medication-related SAEs and the number of SAEs that were not related to the trial medication will be provided in Table 3. The chi-squared test will be used to compare the safety outcomes between the treatment arms attributable to the trial medication.

### Additional analyses

Besides the IIT, we will perform a mITT. In case of lack of compliance or improper conduct of a trial, the inclusion of all the data in the IIT or mITT tends to reduce the effectiveness in a clinical trial since the groups to be compared may look more similar [9]. Therefore, we will also use two alternative FASs of patients to further explore treatment effectiveness—as the FAS might be more appropriate to predict effectiveness in the real world. In addition, to estimate efficacy, we will perform a standard analysis with the two PPSs of patients. However, the effect of compliers assigned to UDCA should be somehow compared with the effect of compliers assigned to placebo, excluding compliers on placebo who would not have been compliant on UDCA, as proposed by Rozenkranz [9]. Therefore, both analyses with the PPSs will be repeated with an exploratory logistic regression on having symptomatic gallstone disease taking into account predicted UDCA compliance in the placebo arm. The results of the above-mentioned data sets will be provided in a supplementary appendix, and any differences between the analyses will be subject of discussion and interpretation.

Stratified randomisation by the presence of gallstone disease at baseline and type of surgery was done to prevent bias while estimating the treatment effect of UDCA on symptomatic gallstone disease. An exploratory logistic regression with interaction terms of treatment with each of the stratification factors will be performed to see if UDCA outcomes may differ by subgroup.

## Discussion

The aim of the UPGRADE trial is to provide evidence regarding the prophylactic use of UDCA in preventing symptomatic gallstone disease after bariatric surgery. This SAP describes the analyses to be published in the primary paper. By publishing this SAP before the data are available, we stimulate transparency of scientific conduct and support reproducibility, thereby allowing others to review our plans.

Randomisation in the UPGRADE trial was stratified for the presence of gallstones. Approximately 20% of patients undergoing bariatric surgery already have asymptomatic gallstones [10]. The majority of earlier performed studies excluded these patients, but we decided to include them because they receive no other or extra treatment after surgery in the current practice. If these patients have a higher risk of becoming symptomatic after surgery, they will be distributed equally over the two study arms.

After adding SG to the inclusion criteria, randomisation was also stratified for the type of surgery. Due to this addition, external validity was increased and internal validity was ensured by incorporating ‘type of surgery’ as a stratification factor. Although the mechanisms of weight loss differ between RYGB and SG, similar percentages of gallstone disease have been reported [11]. To conform to the guidelines of Good Clinical Practice, we decided that patients—who did undergo SG before the protocol amendment was approved—were excluded from the trial and did not receive trial medication. Furthermore, the conduct of the trial forced us to perform the stratified randomisation before the patient underwent surgery. Consequently, a minority of patients was allocated to the wrong type of surgery. Most of them received a SG instead of a RYGB, because the RYGB was technically not possible. These patients will be excluded from the PPSs, because of a protocol deviation.

In our trial, patients were not monitored and cared for more considerately than patients treated outside the trial. Furthermore, we did not stimulate therapy compliance by frequent positive enforcement, because we aimed to provide a realistic manifestation of daily clinical practice. However, we did decide to extend the period in which patients had to start with the trial medication, from 2 weeks to a maximum of 8 weeks after surgery during the course of the trial. We will investigate whether the timing of the start of the trial medication influences the efficacy of UDCA.

We are aware that many studies have reported poor compliance of UDCA after bariatric surgery. Consequently, there is a chance that the expected attrition rate will be exceeded. If this is the case, we assume that we will not need a higher number of patients to assure the availability of sufficient evaluable patients, because the relative reduction is probably larger than the 50% reduction used in the power analysis.

In our trial, the postoperative ultrasound is performed during a regular follow-up visit at the outpatient clinic. Due to the unforeseen and unique bankruptcy of two participating centres, and the coronavirus disease (COVID-19) pandemic, the regular follow-up schedule was suspended temporarily. Therefore, we decided to maintain a relatively large window (18 to 30 months) in which the postoperative ultrasound—originally scheduled at 24 months—has to be carried out. We will explore whether the contrast between the two study arms is stable across different time frames in order to assess the impact of the logistic artefact.

The results of the UPGRADE trial will optimise the postoperative management of bariatric patients and probably lead to a reduction of patients with symptomatic gallstone disease. This trial has the potential to affect daily clinical practice and improve patient outcomes. Second, cost-effectiveness, cost-utility and budget impact analyses of postoperative UDCA prophylaxis may demonstrate the societal value of prophylactic UDCA treatment for health care providers, insurers and taxpayers.

## Supplementary information

**Additional file 1: Figure S1.** Number of participants in additional distinct analyses.

**Additional file 2: Table S1.** SAP Guidance Document: Recommended Items to Address in a Clinical Trial SAP^a^ [1].

## Data Availability

Not applicable.

## Abbreviations

AEs: Adverse events
BMI: Body mass index
CONSORT: Consolidation Standard of Reporting Trials
EQ-5D-5L: EuroQol 5 Dimensions 5 Levels
FAS: Full analysis set
IRB: Institutional review board
ITT: Intention-to-treat
mITT: Modified intention-to-treat
PPS: Per-protocol set
RYGB: Roux-en-Y gastric bypass
SAEs: Serious adverse events
SAP: Statistical analysis plan
SG: Sleeve gastrectomy
SUSARs: Suspected unexpected serious adverse reactions
UDCA: Ursodeoxycholic acid
