# Supplementary material for: Ursodeoxycholic acid for the prevention of symptomatic gallstone disease after bariatric surgery: statistical analysis plan for a randomised controlled trial (UPGRADE trial)
Source: Trials. 2020 Jul 23;21:676. doi: 10.1186/s13063-020-04605-7 (PMC7376318; doi:10.1186/s13063-020-04605-7)
Supplement: Supplementary file 1 — Additional file 1: Figure S1. Number of participants in additional distinct analyses. [file 13063_2020_4605_MOESM1_ESM.docx]

**Supplemental Figure 1. Number of participants in additional distinct analyses**

## Allocation

**Allocated to UDCA (n= )**

**Allocated to placebo (n= )**

## Analyses

**Analysed in ITT excluding protocol violations (n= )**

**Analysed in ITT excluding protocol violations (n= )**

**Analysed in mITT (n= )**

- Excluded from analysis (n= )

**Analysed in mITT (n= )**

- Excluded from analysis (n= )

**Analysed in FAS-1 (n= )**

- Excluded from analysis (n= )

**Analysed in FAS-2 (n= )**

- Excluded from analysis (n= )

**Analysed in FAS-1 (n= )**

- Excluded from analysis (n= )

**Analysed in FAS-2 (n= )**

- Excluded from analysis (n= )

**Analysed in PPS-1 (n= )**

- Excluded from analysis (n= )

**Analysed in PPS-2 (n= )**

- Excluded from analysis (n= )

**Analysed in PPS-1 (n= )**

- Excluded from analysis (n= )

**Analysed in PPS-2 (n= )**

- Excluded from analysis (n= )
