# Supplementary material for: Ursodeoxycholic acid for the prevention of symptomatic gallstone disease after bariatric surgery: statistical analysis plan for a randomised controlled trial (UPGRADE trial)
Source: Trials. 2020 Jul 23;21:676. doi: 10.1186/s13063-020-04605-7 (PMC7376318; doi:10.1186/s13063-020-04605-7)
Supplement: Supplementary file 2 — Additional file 2: Table S1. SAP Guidance Document: Recommended Items to Address in a Clinical Trial SAPa [1]. [file 13063_2020_4605_MOESM2_ESM.docx]

**Table. SAP Guidance Document: Recommended Items to Address in a Clinical Trial SAP^a^ [1]**

**Section 1: Administrative Information Page(s): Row(s):**

Title and trial registration

1a Descriptive title that matches the protocol, with SAP either as a forerunner or subtitle, and trial acronym (if applicable) 1 1-3

1b Trial registration number 1 22

SAP version

2 SAP version number with dates 1 24

Protocol version

3 Reference to version of protocol being used 1 25

SAP revisions

4a SAP revision history 1 26

4b Justification for each SAP revision 1 27

4c Timing of SAP revisions in relation to interim analyses, etc NA

Roles and responsibility

5 Names, affiliations, and roles of SAP contributors 1 10-17

15 384-387

Signatures of:

6a Person writing the SAP NA

6b Senior statistician responsible NA

6c Chief investigator/clinical lead NA

**Section 2: Introduction**

Background and rationale

7 Synopsis of trial background and rationale including a brief description of research question and brief justification for 3 57-68

undertaking the trial

Objectives

8 Description of specific objectives or hypotheses 3 57-68

**Section 3: Study Methods**

Trial design

9 Brief description of trial design including type of trial (eg, parallel group, multiarm, crossover, factorial) and allocation 3 71-79

ratio and may include brief description of interventions

Randomization **Page(s): Row(s):**

10 Randomization details, eg, whether any minimization or stratification occurred (including stratifying factors used 3-4 78-83

or the location of that information if it is not held within the SAP)

Sample size

11 Full sample size calculation or reference to sample size calculation in protocol (instead of replication in SAP) 4 95

Framework

12 Superiority, equivalence, or noninferiority hypothesis testing framework, including which comparisons will be 3 71-73

presented on this basis (Superiority framework not explicitly mentioned)

Statistical interim analyses and stopping guidance

13a Information on interim analyses specifying what interim analyses will be carried out and listing of time points NA

13b Any planned adjustment of the significance level due to interim analysis NA

13c Details of guidelines for stopping the trial early NA

Timing of final analysis

14 Timing of final analysis, eg, all outcomes analyzed collectively or timing stratified by planned length of follow-up 5 117-118

Timing of outcome assessments

15 Time points at which the outcomes are measured including visit “windows” 4 83-95

**Section 4: Statistical Principles**

Confidence intervals and P values

16 Level of statistical significance 5 120-121

17 Description and rationale for any adjustment for multiplicity and, if so, detailing how the type 1 error is to be controlled NA

18 Confidence intervals to be reported 5 120-121

Adherence and protocol deviations

19a Definition of adherence to the intervention and how this is assessed including extent of exposure 5 128-133

9-10 235-248

19b Description of how adherence to the intervention will be presented 10 245-248

Table 3

19c Definition of protocol deviations for the trial 6 138-144

19d Description of which protocol deviations will be summarized NA

Analysis populations

20 Definition of analysis populations, eg, intention to treat, per protocol, complete case, safety 6 138-160

11 276-283

**Section 5: Trial Population Page(s): Row(s):**

Screening data

21 Reporting of screening data (if collected) to describe representativeness of trial sample Figure 1

Eligibility

22 Summary of eligibility criteria 3 73-78

Recruitment

23 Information to be included in the CONSORT flow diagram Figure 1

Withdrawal/follow-up

24a Level of withdrawal, eg, from intervention and/or from follow-up 6 133-134

Figure 1

24b Timing of withdrawal/lost to follow-up data NA

24c Reasons and details of how withdrawal/lost to follow-up data will be presented 6 133-134

Figure 1

Baseline patient characteristics

25a List of baseline characteristics to be summarized 7 179-185

25b Details of how baseline characteristics will be descriptively summarized 7-8 185-192

Table 1

**Section 6: Analysis**

Outcome definitions

List and describe each primary and secondary outcome including details of:

26a specification of outcomes and timings. If applicable include the order of importance of primary or key secondary end 8-11 195-273

points (eg, order in which they will be tested)

26b specific measurement and units (eg, glucose control, hbA1c [mmol/mol or %]) 8-11 195-273

26c any calculation or transformation used to derive the outcome (eg, change from baseline, QoL score, time to event, 8-11 195-273

logarithm, etc)

Analysis methods

27a what analysis method will be used and how the treatment effects will be presented 8 202-206

Table 2

27b any adjustment for covariates See additional analyses

27c methods used for assumptions to be checked for statistical methods NA

27d details of alternative methods to be used if distributional assumptions do not hold, eg, normality, proportional NA

hazards, etc

27e any planned sensitivity analyses for each outcome where applicable See additional analyses

27f any planned subgroup analyses for each outcome including how subgroups are defined 6 138-160

Missing data

28 Reporting and assumptions/statistical methods to handle missing data (eg, multiple imputation) 7 163-176

Additional analyses

29 Details of any additional statistical analyses required, eg, complier-average causal effect^10^ analysis 11-12 286-302

Harms

30 Sufficient detail on summarizing safety data, eg, information on severity, expectedness, and causality; 11 276-283

details of how adverse events are coded or categorized; how adverse event data will be analyzed, ie,

grade 3/4 only, incidence case analysis, intervention emergent analysis

Statistical software

31 Details of statistical packages to be used to carry out analyses 5 119-120

References

32a References to be provided for nonstandard statistical methods NA

32b Reference to Data Management Plan NA

32c Reference to the Trial Master File and Statistical Master File NA

32d Reference to other standard operating procedures or documents to be adhered to NA

Abbreviations: CONSORT, Consolidated Standards of Reporting Trials; hbA1c, hemoglobin A1c; QoL, quality of life; SAP, statistical analysis plan.

^a^Reproduced with permission from the author

1. Gamble C, Krishan A, Stocken D, Lewis S, Juszczak E, Doré C, et al. Guidelines for the Content of Statistical Analysis Plans in Clinical Trials. Jama. 2017;318(23):2337-43.
